# Risk factors for early and delayed post-operative bleeding after endoscopic submucosal dissection of gastric neoplasms, including patients with continued use of antithrombotic agents

**DOI:** 10.1186/1471-230X-14-172

**Published:** 2014-10-03

**Authors:** Tomoaki Matsumura, Makoto Arai, Daisuke Maruoka, Kenichiro Okimoto, Shoko Minemura, Hideaki Ishigami, Keiko Saito, Tomoo Nakagawa, Tatsuro Katsuno, Osamu Yokosuka

**Affiliations:** Department of Gastroenterology and Nephrology, Graduate School of Medicine, Chiba University, Inohana 1-8-1, Chiba-City, 260-8670 Japan

**Keywords:** Endoscopic submucosal dissection, ESD, Antithrombotic agents, Post-operative bleeding, Gastric neoplasms, Risk factor

## Abstract

**Background:**

Endoscopic submucosal dissection (ESD) has become widely accepted as a standard treatment for gastric epithelial neoplasms. Antithrombotic agents are widely used to prevent thromboembolic disease. However, the feasibility of endoscopic procedures for patients using such agents has been rarely investigated. The aim of this study was to identify risk factors for post-operative bleeding after gastric ESD and to evaluate the relationship between the use of antithrombotic agents and post-operative bleeding.

**Methods:**

From June 2005 to March 2014, 413 patients with 425 gastric neoplasms were treated by ESD. The demographic and clinical parameters associated with post-operative bleeding were investigated. 83 patients receiving antithrombotic agents were separately assessed using various methods of administration during the ESD procedure. Post-operative bleeding that occurred within 5 days of ESD was defined as early post-operative bleeding, whereas subsequent bleeding was defined as delayed bleeding.

**Results:**

The overall post-operative bleeding rate was 4.7%. In patients with continued low-dose aspirin (LDA), heparin replacement (HR), or continued LDA along with HR, post-operative bleeding rates were 9.5%, 23.8%, and 25.0%, respectively. On multivariate analysis, a specimen size of ≥40 mm was a risk factor for early post-operative bleeding [odds ratio (OR) 6.08, 95% CI: 1.74–21.27], and HR and chronic kidney disease (CKD) requiring hemodialysis were risk factors for delayed bleeding (OR 12.23, 95% CI: 2.63–56.77 and OR 28.35, 95% CI: 4.67–172.11, respectively). Continued LDA was not a risk factor for post-operative bleeding.

**Conclusions:**

Large specimen size is a risk factor for early post-operative bleeding, and HR and CKD requiring hemodialysis are risk factors for delayed bleeding. Patients with risk factors should be carefully watched, allowing for the timing of post-operative bleeding after ESD.

## Background

Endoscopic submucosal dissection (ESD) is widely recognized as the optimal treatment for gastric epithelial neoplasms [1–4]. The safety of gastric ESD has been mostly established; however, post-operative bleeding is still the main complication affecting the outcome of the procedure.

Antithrombotic agents are widely used to prevent thromboembolic disease. The number of patients taking antithrombotic agents has increased as a result of increases in the incidence of ischemic heart disease, cerebrovascular disease, and arteriosclerosis obliterans. Although antithrombotic agents are very effective in the management of thromboembolic diseases [5, 6], their use increases the incidence of gastrointestinal (GI) bleeding [7–9]. MacQuaid et al. found that low-dose aspirin (LDA) increases the risk of major GI bleeding (relative risk 2.07, 95% CI: 1.61–2.66) in meta-analysis [8]. In addition, Lanas et al. reported that *Helicobacter pylori* infection increased the risk of upper GI bleeding in patients taking LDA [10]. On the other hand, endoscopic procedures such as ESD cause post-operative bleeding at a constant rate. Previous studies have reported that the post-operative bleeding rate was approximately 5% after gastric ESD [11–14]. Because many patients who undergo gastric ESD are infected with *Helicobacter pylori*, the post-ESD bleeding rate for patients receiving antithrombotic agents is expected to be >5%.

In clinical practice, healthcare workers need to balance the risks of hemorrhage after endoscopic procedures and thromboembolic events after medication cessation. The American Society for Gastrointestinal Endoscopy (ASGE) guidelines for the management of antithrombotic agents for endoscopic procedures recommend the continued use of LDA for GI endoscopies, even for procedures with a high risk of hemorrhage [15]. In contrast, the European Society of Gastrointestinal Endoscopy (ESGE) guidelines recommend that LDA should be continued for most endoscopies, but discontinued for 5 days prior to ESD and other procedures with a high risk of hemorrhagic complications, provided the risk of thromboembolic events is low [16]. Guidelines for the management of anticoagulant and antiplatelet therapy in patients undergoing endoscopy were published in Japan in 2012 [17, 18]. The Japanese guidelines recommend endoscopic procedures without interruption of LDA therapy in patients at high risk of thromboembolic events who use LDA alone. On the other hand, it is recommended that anticoagulants, such as warfarin, can be replaced by heparin as a bridge therapy [17, 18]. However, there is insufficient data to support these strategies. Furthermore, the risk of bleeding after gastric ESD for patients routinely using LDA remains controversial [19, 20]. The aim of the present study was to identify risk factors for post-operative bleeding and to evaluate the relationship between the use of antithrombotic agents and post-operative bleeding in patients who underwent ESD for gastric neoplasms.

## Methods

### Patients

This was a retrospective study. A total of 425 gastric epithelial neoplasms (412 early gastric cancers and 13 gastric adenomas) in 413 patients were treated with ESD at the Chiba University Hospital (Japan) from June 2005 to March 2014. Of the 413 patients, 83 (20.0%) were on antithrombotic therapy (warfarin potassium in 21 patients, antiplatelet drugs in 62 patients, both warfarin potassium and antiplatelet drugs in 4 patients, and two kinds of antiplatelet drug in 7 patients). All 83 patients were classified into one of two groups, at higher- or lower-risk of thromboembolism, according to risk stratifications based on published criteria [17, 18]. The high risk of thromboembolism was defined based on the following: two months following coronary artery bare metal stenting; twelve months following coronary artery drug eluting stenting; two months following carotid arterial revascularization (carotid endarterectomy or stenting); ischemic stroke or transient ischemic attack with >50% stenosis of major intracranial arteries; recent ischemic stroke or transient ischemic attack; obstructive peripheral artery disease ≥ Fontaine grade 3 (rest pain); ultrasonic examination of carotid arteries and magnetic resonance angiography of the head and neck region where withdrawal is considered high risk of thromboembolism; history of cardiogenic brain embolism; atrial fibrillation accompanying valvular heart disease; atrial fibrillation without valvular heart disease but with a high risk of stroke; following mechanical mitral valve replacement; history of thromboembolism following mechanical valve replacement; antiphospholipid antibody syndrome; and deep vein thrombosis/pulmonary thromboembolism [17, 18]. Of the 46 high-risk patients, 21 continued LDA during the procedures, 21 underwent intravenous heparin replacement (HR), and 4 patients continued LDA and underwent HR. In the 37 low-risk patients, antithrombotic agents were temporarily stopped before the procedures and restarted within 5 days of the procedures (day 1, 3, and 5 after procedures in 26, 2, and 9 patients, respectively).

The indications for ESD were determined on the basis of endoscopic findings, including chromoendoscopy with indigo carmine dye, and biopsy. We referred to the criteria described by Gotoda et al. [1] for possible node-negative gastric epithelial neoplasia.

### ESD procedure

ESD was performed with a conventional single-channel endoscope (GIF-H260Z or -Q260J; Olympus, Tokyo, Japan) or a two-channel endoscope (2TQ260 M; Olympus). We mainly used an IT knife 2 (KD-611 L; Olympus), and electrosurgical current was applied using an electrosurgical generator (VIO 300D; ERBE, T¨ubingen, Germany). In addition, we used other electrosurgical knives as necessary, including an IT knife (KD-610 L or KD-611 L; Olympus), a Hook knife (KD-620LR; Olympus), or a Flush knife (DK-2618; Fujifilm Inc., Tokyo, Japan), with an ICC200 (ERBE) electrosurgical generator. The injection solutions contained glycerin and hyaluronic acid sodium (0.4%) with 1% indigo carmine dye. The ulcers that developed after ESD were endoscopically examined and any visible vessels were heat-coagulated using hemostatic forceps (FD-410LR; Olympus). The resected specimens were stretched, pinned flat on a corkboard, and measured.

### Management after ESD

Beginning on the day of ESD, omeprazole (40 mg/day) was administered intravenously. From June 2005 to September 2013, we performed second-look endoscopies the day after ESD and coagulated all exposed vessels on artificial ulcers, regardless of whether or not bleeding was present. After October 2013, we did not routinely perform second-look endoscopies. We also checked hemoglobin levels the morning after ESD. If no ESD-related complications occurred, patients were allowed to have a liquid diet. From 2 days after ESD, all patients received 20-mg omeprazole or esomeprazole, and sodium alginate (120 ml/day) orally. Patients were discharged the day after a diet was established. After discharge, patients were instructed to take 20-mg omeprazole or esomeprazole once a day for 28 days.

### Management of patients taking anticoagulants

When gastric ESD was performed in patients receiving oral anticoagulants such as warfarin, anticoagulants were stopped temporarily 3 days before the procedure and HR was used as a bridge therapy. Unfractionated heparin was used for HR. A continuous administration of heparin was initiated and controlled to keep the activated partial thromboplastin time (APTT) at approximately 60 s. Intravenous HR was stopped 6 h before ESD. In addition, the prothrombin time international normalized ratio (PT-INR) was measured before ESD to confirm that the drug effect had disappeared. After ESD, intravenous HR was restarted 2 h after the procedure and oral anticoagulants were restarted the next day after the procedure. Heparin sodium was discontinued when the PT-INR level had reached approximately 1.50.

### Post-operative bleeding

Post-operative bleeding was defined as a decrease in the blood hemoglobin level of >2 g/dL accompanied by an occurrence of hematemesis, melena, or a combination of unstable vital signs within 4 weeks of ESD. The typical period of hospitalization after ESD was 5 days. Therefore, post-operative bleeding that occurred within 5 days of ESD was defined as early post-operative bleeding, and subsequent bleeding was defined as delayed post-operative bleeding. All patients with post-operative bleeding underwent emergency esophagogastroduodenoscopy (EGD). Information was recorded regarding the time of bleeding and whether any interventions were performed.

### Histopathological examination and curability after ESD

Histopathological examination was based on the 2010 Japanese Classification of Gastric Carcinoma issued by the Japanese Gastric Cancer Association (JGCA) [21]. The entire resected specimen was cut into parallel 2-mm sections and examined with hematoxylin and eosin staining for detailed analysis, including the analysis of the deepest portion containing infiltrating cancer cells. Gastric carcinomas were classified as differentiated or undifferentiated. The former type included well-differentiated tubular adenocarcinoma, moderately differentiated tubular adenocarcinoma, and papillary adenocarcinoma. The latter type included poorly differentiated adenocarcinoma, signet ring cell carcinoma, and mucinous adenocarcinoma. *En bloc* resection was defined as resection in a single piece. Complete resection was defined as an *en bloc* resection of a tumor that was shown to be free of cancer cells at both the horizontal and vertical cut ends. The resection was judged as curative when all the following criteria were met: *en bloc* removal, tumor size ≤ 2 cm, differentiated type, pT1a, negative horizontal margin (HM0), negative vertical margin (VM0), and no lymphovascular infiltration [ly (−), v (−)]. Curative resection for early gastric carcinomas that fell under the expanded indications was defined as follows: *en bloc* resection, HM0, VM0, ly (−), and v (−) as well as (1) tumor size > 2 cm, differentiated type, pT1a, and ulceration (UL) −; (2) tumor size ≤ 3 cm, differentiated type, pT1a, and UL +; (3) tumor size ≤ 2 cm, undifferentiated type, pT1a, and UL −; or (4) tumor size ≤ 3 cm, differentiated type, and pT1b (SM1, ≤0.5 mm from the muscularis mucosae) [22].

### Statistical analysis

Some of the patients had more than one gastric neoplasm, and therefore underwent more than one ESD procedure. For statistical purposes, the data from multiple ESD-induced ulcers occurring in certain patients were assumed to constitute independent observations. Baseline data are presented as mean ± SD. Differences in clinical parameter values between groups were analyzed by unpaired *t*-tests or chi-square tests. The post-operative bleeding rate was established, and the factors associated with post-operative bleeding were assessed using univariate and multivariate analyses. Variables with a *P*-value <0.1 on univariate analysis were entered into multivariate logistic regression analysis. All statistical analyses were performed using SPSS 20.0 (SPSS Inc., Chicago, IL, USA). A *P*-value of <0.05 was considered statistically significant.

### Ethics considerations

Informed consent was obtained from all patients. The study was conducted in accordance with the Declaration of Helsinki (1995) after the protocol had been approved by the institutional review board of the Chiba University School of Medicine. In addition, this study was registered at the University Hospital Medical Information Network (UMIN000013241).

## Results

### Demographic data

Clinical outcomes are shown in Table 1. The complete *en bloc* resection rate was 95.1%, perforation rate was 0.5%, and overall post-operative bleeding rate was 4.7%. The clinical characteristics of gastric lesions in patients with gastric neoplasms are shown in Table 2.

**Table 1:** **Clinical outcomes of ESD**

Characteristic	
Complete *en bloc* resection (%)	95.1
Curability	
Curative resection (%)	72.3
Expanded curative resection (%)	21.4
Non-curative resection (%)	6.3
Complication	
Perforation (%)	0.5
Post-operative bleeding (%)	4.7

**Table 2:** **Clinical characteristics of gastric lesions in patients with gastric neoplasms**

	Total n = 425	Post-operative bleeding n = 20	No post-operative bleeding n = 405	*p*-value
Sex (male/female)	302/123	18/2	284/121	n. s.*
Age (years, mean ± SD)	72.1 ± 8.6	71.9 ± 10.0	72.1 ± 8.5	n. s.**
Tumor location (n, %)				
Upper	65 (15)	3 (15)	62 (15)	n. s.*
Middle	179 (42)	6 (30)	173 (43)	n. s.*
Lower	181 (43)	11 (55)	170 (42)	n. s.*
Tumor size (mm, mean ± SD)	18.4 ± 12.6	29.6 ± 21.8	17.8 ± 11.7	<0.05**
Specimen size (mm, mean ± SD)	33.9 ± 13.3	41.3 ± 17.6	33.6 ± 12.9	0.07**
Invasion depth				
M	396	18	378	n. s.*
SM1	15	1	14	n. s.*
SM2	14	1	13	n. s.*
Ulceration (+/-)	418/7	19/1	399/6	n. s.*
Medication used (n, %)				
Continued use of LDA	21 (9)	2 (10)	19 (5)	n. s.*
Continued use of LDA + heparin replacement	4 (1)	1 (5)	3 (0.7)	n. s.*
Heparin replacement	21 (5)	5 (25)	16 (4)	<0.05*
Cessation of antithrombotic agent during ESD procedure	41 (9)	2 (10)	39 (10)	n. s.*
Comorbidity (n, %)				
Liver cirrhosis	20 (5)	2 (10)	18 (4)	n. s.*
CKD undergoing hemodialysis	20 (5)	4 (20)	16 (4)	<0.05*
Second-look endoscopy (+/-)	356/69	19/1	337/68	n. s.*

LDA: Low-dose aspirin.

CKD: Chronic kidney disease.

*Chi-square test.

**Unpaired *t*-test.

n.s.: not significant.

### Post-operative bleeding

Twenty patients (4.7%) had post-operative bleeding. Table 2 shows clinical differences between patients with post-operative bleeding and those without. In patients with continued LDA, HR, or continued LDA and HR, the post-operative bleeding rates were 9.5%, 23.8%, and 25.0%, respectively. In patients with chronic kidney disease (CKD) requiring hemodialysis, the post-operative bleeding rate was 20%. Among all patients with post-operative bleeding patients with HR and CKD requiring hemodialysis were significantly more likely to bleed than those without post-operative bleeding (*P* < 0.05). In addition, the maximum diameter of the tumor was significantly larger in patients with post-operative bleeding than those without post-operative bleeding (*P* < 0.05). All the post-operative bleeding occurred within 10 days of ESD. Early and delayed post-operative bleeding occurred in 11 and 9 patients, respectively. In 3 patients, we confirmed post-operative bleeding on second-look endoscopy, and two of them were patients taking antithrombotic agents.

### Factors associated with post-operative bleeding

**Figure 1:**
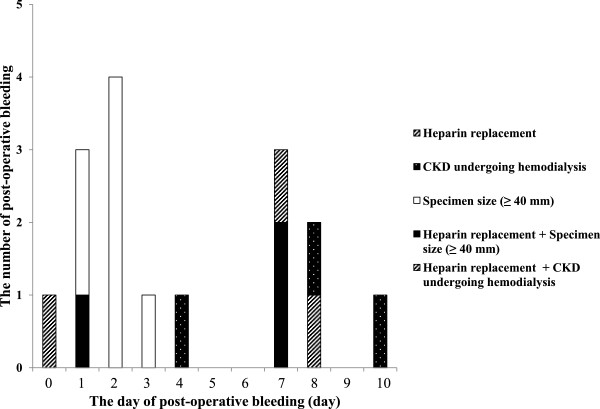
**The relationship between the time of post-ESD bleeding and risk factors:** **most cases of** **post-operative** **bleeding in patients with specimen size ≥ 40 mm occurred within 5 days of ESD.** On the other hand, most cases of post-operative bleeding in patients with one or more risk factors occurred after 6 days after ESD.

Table 3 shows univariate and multivariate analyses of the risk factors associated with overall post-operative bleeding. These analyses revealed that HR, CKD requiring hemodialysis, and a maximum specimen diameter of ≥40 mm were risk factors for post-operative bleeding [odds ratio (OR) 5.77, 95% CI: 1.67–19.96; OR 33.86, 95% CI: 4.72–242.74; and OR 3.70, 95% CI: 1.09–12.52, respectively]. We repeated the univariate analysis, changing the threshold for specimen size from 30 to 50 mm in 10 mm increments. We determined the threshold when the probability value was the lowest and found a specimen size threshold of 40 mm. The maximum diameter of tumor size was strongly correlated to specimen size (Pearson product moment correlation, *r* = 0.68, *P* < 0.05). Therefore, tumor size was excluded from this analysis. The relationship between risk factors and the time of post-operative bleeding is shown in Figure 1. Table 4 shows the univariate analysis of the risk factors associated with early post-operative bleeding. This analysis revealed that a specimen size of ≥40 mm was a risk factor for early post-operative bleeding (OR 6.08, 95% CI: 1.74–21.27). In contrast, Table 5 shows the univariate and multivariate analyses of the risk factors associated with delayed post-operative bleeding. These analyses revealed that HR and CKD requiring hemodialysis were risk factors for delayed post-operative bleeding (OR 12.23, 95% CI: 2.63–56.77 and OR 28.35, 95% CI: 4.67–172.11, respectively). The post-operative bleeding rate was higher in patients with continued LDA. However, there was no statistical association between post-operative bleeding and continued LDA.

**Table 3:** **Univariate and multivariate analyses of risk factors associated with overall post-operative bleeding**

	Univariate analysis		Multivariate analysis	
Risk factor	OR (95% CI)	*p*-value	OR (95% CI)	*p*-value
Male gender (vs. Female)	3.91 (0.89-17.11)	0.07*	6.40 (0.69-58.69)	0.10
Age ≥70 years (vs. ≤69 years)	1.01 (0.39-2.58)	0.99		
Tumor location Lower (vs. Upper and Middle)	1.65 (0.67-4.08)	0.27		
Continued use of LDA	2.97 (0.81-10.92)	0.10		
Continued use of LDA + heparin replacement	6.84 (0.68-68.89)	0.10		
Heparin replacement	7.99 (2.77-22.99)	<0.01*	5.77 (1.67-19.96)	0.01*
Cessation of antithrombotic agent during ESD procedure (vs. No antithrombotic therapy)	1.82 (0.38-8.68)	0.45		
Liver cirrhosis	1.51 (0.33-6.83)	0.59		
CKD undergoing hemodialysis	19.40 (4.75-79.19)	<0.01*	33.86 (4.72-242.74)	<0.01*
Specimen size ≥40 mm (vs. < 40 mm)	2.84 (1.14-7.09)	0.03*	3.70 (1.09-12.52)	0.04*
Procedure time ≥90 min (vs. < 90 min)	3.05 (1.12-8.29)	0.03*	2.45 (0.74-8.16)	0.15
Second-look endoscopy (vs. No second-look endoscopy)	3.83 (0.51-29.14)	0.19		

OR: Odds ratio.

CI: Confidence interval.

LDA: Low-dose aspirin.

CKD: Chronic kidney disease.

*p<0.05.

**Table 4:** **Univariate and multivariate analyses of risk factors associated with early post-operative bleeding**

	Univariate analysis		Multivariate analysis	
Risk factor	OR (95% CI)	*p*-value	OR (95% CI)	*p*-value
Male gender (vs. Female)	4.25 (0.53-33.61)	0.17		
Age ≥70 years (vs. ≤69 years)	1.45 (0.38-5.57)	0.58		
Tumor location Lower (vs. Upper and Middle)	2.36 (0.68-8.20)	0.17		
Continued use of LDA	3.66 (0.74-17.93)	0.11		
Continued use of LDA + heparin replacement	13.30 (1.27-139.25)	0.03*	32.88 (2.61-414.05)	<0.01*
Heparin replacement	3.50 (0.71-17.10)	0.12		
Cessation of antithrombotic agent during ESD procedure (vs. No antithrombotic therapy)	3.08 (0.60-15.87)	0.18		
Liver cirrhosis	1.33 (0.16-10.81)	0.78		
CKD undergoing hemodialysis	4.92 (0.56-43.20)	0.15		
Specimen size ≥40 mm (vs. < 40 mm)	6.08 (1.74-21.27)	<0.01*	8.03 (2.03-31.72)	<0.01*
Procedure time ≥90 min (vs. < 90 min)	3.44 (0.81-14.71)	0.10		
Second-look endoscopy (vs. No second-look endoscopy)	1.96 (0.24-15.60)	0.52		

OR: Odds ratio.

CI: Confidence interval.

LDA: Low-dose aspirin.

CKD: Chronic kidney disease.

*p<0.05.

**Table 5:** **Univariate and multivariate analyses of risk factors associated with delayed post-Operative bleeding**

	Univariate analysis		Multivariate analysis	
Risk factor	OR (95% CI)	*p*-value	OR (95% CI)	*p*-value
Male gender (vs. Female)	3.38 (0.41-27.32)	0.25		
Age ≥70 years (vs. ≤69 years)	0.67 (0.17-2.53)	0.55		
Tumor location Lower (vs. Upper and Middle)	1.05 (0.28-3.99)	0.55		
Continued use of LDA	1.98 (0.23-16.4)	0.52		
Continued use of LDA + heparin replacement	N/A	N/A		
Heparin replacement	13.89 (3.48-55.39)	<0.01*	12.23 (2.63-56.77)	<0.01*
Cessation of antithrombotic agent during ESD procedure (vs. No antithrombotic therapy)	N/A	N/A		
Liver cirrhosis	1.67 (0.20-13.9)	0.63		
CKD undergoing hemodialysis	33.16 (6.67-164.82)	<0.01*	28.35 (4.67-172.11)	<0.01*
Specimen size ≥40 mm (vs. < 40 mm)	0.92 (0.19-4.55)	0.92		
Procedure time ≥90 min (vs. < 90 min)	2.56 (0.67-9.78)	0.16		
Second-look endoscopy (vs. No second-look endoscopy)	N/A	N/A		

OR: Odds ratio.

CI: Confidence interval.

LDA: Low-dose aspirin.

CKD: Chronic kidney disease.

N/A: Not applicable.

*p<0.05.

### Management of post-operative bleeding

All patients with post-operative bleeding underwent emergency EGD and endoscopic treatment and recovered completely after endoscopic and medical treatment.

## Discussion

In previous guidelines, the cessation of antithrombotic agents during endoscopic procedures was recommended because the emphasis was on the prevention of GI bleeding. However, recently the risks associated with the cessation of such agents have been widely reported [23–26]. Therefore, the management of antithrombotic agents during endoscopic procedures has changed. In Japan, the guidelines were revised in 2012, and endoscopic procedures can be performed without the interruption of LDA therapy in patients at high risk for thromboembolic events [17, 18]. However, no consensus has been reached and controversy exists as to whether the use of these agents is a risk factor for post-operative bleeding after ESD. In previous studies, Koh et al. and Takeuchi et al. reported that the use of antithrombotic drugs was a risk factor for post-operative bleeding after ESD [27, 28]. However, in these reports, antithrombotic drug therapies were interrupted preoperatively in all patients. In this regard, these studies differed from our research that focused on the continued use of antithrombotic agents and risk factors for post-operative bleeding. On the other hand, Cho et al. also reported an increased risk of bleeding after gastric ESD in a recent study of 19 patients with the continued use of LDA [19]. In contrast, Sanomura et al. reported that the continued use of LDA did not increase the risk of bleeding during or after ESD in 25 patients with the continued use of LDA [20]. In our study, we studied 83 patients and used various methods for administering antithrombotic agents. Our study included 25 patients with the continued use of LDA. The continued use of LDA was not a risk factor but HR, CKD requiring hemodialysis, and a maximum specimen diameter of ≥40 mm were risk factors for post-operative bleeding. In addition, in our study, we evaluated the relationship between risk factors and the time of post-operative bleeding. Large specimen size was a risk factor for early post-operative bleeding, and HR and CKD requiring hemodialysis were risk factors for delayed bleeding. A temporary cessation of antithrombotic agents during the ESD procedure was not a risk factor for post-operative bleeding.

In previous studies, Kakushima et al. reported that ESD-induced gastric ulcers healed within 8 weeks, regardless of the size and location, and fibrosis and wall thickening were observed from 2 weeks after ESD [29, 30]. In addition, Goto et al. reported that visible vessels, which occurred in approximately one quarter of the ulcers within 3 days of ESD, were rarely observed 4 days after ESD [31]. Considering these studies and our results, these visible vessels may cause early post-operative bleeding and may depend on the size of ESD-induced ulcers. In contrast, delayed bleeding may be caused by the delayed healing of ulcers, which in turn may be caused by HR and CKD requiring hemodialysis. Yoshio et al. reported that gastric ESD under HR was associated with a high risk of post-operative bleeding (37.5%), a finding that was similar to the result in our study (23.8%) [32]. These high incidences of post-operative bleeding may be caused using two kinds of anticoagulant therapy (oral anticoagulants and intravenous heparin) from the day after ESD. We routinely performed second-look endoscopy before September 2013. However, in a prospective, randomized, controlled study, Ryu et al. found that second-look endoscopy was not associated with clinical outcomes including bleeding and morbidity after gastric ESD [33]. For this reason, we did not perform second-look endoscopy routinely after October 2013. In the present study, we also analyzed the relationship between second-look endoscopy and post-operative bleeding. There was no statistical association between post-operative bleeding and second-look endoscopy.

There are several limitations to this study. First, the study was a retrospective study and was conducted at a single center. Second, this study contained a small number of cases with the continued use of antithrombotic agents. Third, the number of patients with post-operative bleeding was small. These limitations indicate that our study requires further validation. In addition, the cessation of antithrombotic agents in patients at high risk of thromboembolism is difficult in view of the risks associated with the cessation of such agents. Therefore, we could not perform a randomized controlled study that stratified patients on the basis of the risk of thromboembolism. In the future, further research involving large numbers of patients is warranted.

## Conclusions

Large specimen size is a risk factor for early post-operative bleeding, and HR and CKD requiring hemodialysis are risk factors for delayed bleeding. In contrast, continued LDA is not a risk factor for post-operative bleeding. Patients with risk factors should be carefully watched, allowing for the timing of post-operative bleeding after ESD.

## Abbreviations

ESD: Endoscopic submucosal dissection
CKD: Chronic kidney disease
LDA: Low-dose aspirin
HR: Heparin replacement
EGD: Esophagogastroduodenoscopy
APTT: Activated partial thromboplastin time.
